# Relationship between measures of adiposity and hypertension amongst secondary school adolescents in an urban setting in Cameroon

**DOI:** 10.11604/pamj.2023.46.57.41547

**Published:** 2023-10-17

**Authors:** Loveline Lum Niba, Lifoter Kenneth Navti, Ahmadou Jingi Musa

**Affiliations:** 1 Department of Public Health, The University of Bamenda, Bambili, North West Region, Cameroon,; 2 Nutrition and Health Research Group (NHRG), Bamenda, Cameroon,; 3 Department of Biochemistry, The University of Bamenda, Bambili, North West Region, Cameroon,; 4 Department of Clinical Sciences, The University of Bamenda, Bambili, North West Region, Cameroon

**Keywords:** Measures of adiposity, hypertension, secondary school, adolescents, Bamenda, Cameroon

## Abstract

**Introduction:**

measures of obesity such as body mass index (BMI), waist circumference (WC) and waist-to-height ratio (WHtR) have been shown to be associated with high blood pressure (BP) in children and adolescents. The purpose of this study was to determine the proportion of secondary school adolescents with elevated BP and high BP in relation to some measures of adiposity (BMI, WC, WHtR) and to examine the association between BP and adiposity indices amongst the children.

**Methods:**

the study was an institutional-based cross-sectional study involving 534 adolescents (mean age 15.1 ± 2.3 years) attending 4 secondary schools (2 public and 2 private) in the Bamenda municipality of the North West Region of Cameroon. Anthropometric and BP measurements were carried out following standard procedures. Diagnosis of hypertension in the children was done by obtaining three elevated systolic or diastolic BP readings (BP ≥ 95^th^ percentile for the child's age, sex and height). Linear regression was used to determine the relationship between BP and some measures of adiposity (BMI, WC, WHtR) amongst the children

**Results:**

the prevalence of elevated BP and hypertension amongst the study participants was 33.3% and 33.3% in the BMI-obese children, 25.9% and 25.2% in the WC overweight/obese children and 29.4% and 41.2% in the “high risk” (WHtR ≥ 0.5) children respectively. Body mass index-obese, WC overweight/obese and “high risk” (WHtR ≥ 0.5) children had a significantly (p <0.05) higher mean SBP and DBP compared to their healthy weight counterparts. Linear regression indicated a significant association (p <0.001) between WC (β=0.75; 95% CI = 0.57, 0.92), BMI (β=0.88; 95% CI = 0.49, 1.25) and WHtR (β= 67.08; 95% CI = 45.64, 88.51) with systolic BP for the unadjusted analysis. After adjusting for age, gender and school type, only WC (β= 0.66; 95% CI = (0.43, 0.89) showed a positive significant (p <0.001) relationship with systolic BP.

**Conclusion:**

this study has demonstrated that WC is positively associated with high BP in children and adolescents. Thus, WC can be used in predicting children and adolescents with a high risk of developing high BP in our setting.

## Introduction

Evidence has shown that by 2025, the number of overweight/obese children aged 5 -17 years globally will increase to 335 million [1]. Also, there has been growing evidence on the increasing prevalence of childhood overweight/obesity in the developing and industrialized nations [2-6]. This has been attributed to rapid urbanization, high rates of sedentary lifestyle and unhealthy eating habits [7]. Obesity in children is defined as the accumulation of excess body fat with a body mass index (BMI) of ≥ 95^th^percentile for age and gender [8] Recent reports in Cameroon indicated that the highest prevalence of overweight/obesity was observed among children in the grass field area including the North West Region [3,9] with a higher prevalence in the urban (23%) compared to the semi-urban area (1.9%) [10]. Moreover, central obesity in children have been found to positively correlate with dyslipidemia, hypertension and alterations in the metabolism of blood glucose and T2DM [11]. The persistent rise in the global prevalence of obesity in the pediatric population due to the nutrition and epidemiological transition is becoming worrisome given it is a major risk factor for metabolic syndrome in this vulnerable group [12,13]. Thus, early detection through screening will help in reducing adverse cardiovascular outcomes in early life. For instance, a report in the Center Region of Cameroon indicated that the prevalence of hypertension among primary school pupils in 2019 was 1.6% [14]. Another report in 2021 in children (3 to 19 years) in the Center and West Regions of Cameroon showed a hypertension prevalence of 8.6% and 12.0% in the urban and semi-urban areas respectively [9]. Moreover, a recent systematic review by Noubiap *et al*. [12] reported an elevated BP prevalence of 5.5% among sub-Saharan African children and adolescents with overweight and obesity being a major risk factor for elevated BP among the children. In addition, a systematic review [15] as well as a meta-analysis [16] have shown that childhood BP levels persist into adulthood, with pubertal hypertension being a strong predictor of hypertension in later life [17]. The prevalence of hypertension among obese children and adolescents has been found to be based on the degree of excess weight [18]. Also, evidence has demonstrated that elevated BP in the pediatric population, is associated with pathological changes such as atherosclerosis, left ventricular hypertrophy and an increase in the carotid intima-media thickness [19], with a greater carotid artery intima-media thickness in obese children [20,21] compared to their non-obese counterparts. Studies in children and adolescents have demonstrated the relationship between obesity and using BMI as a proxy [22], WC [23] and WHtR [24] with hypertension. While some reports have found WC measurements [13,23] as the best tool in predicting cardiometabolic risk in children, others have found WHtR [25,26]. However, Millar *et al*. [27] did not find any superiority of the central adiposity indices over BMI in predicting cardio metabolic risk. Despite evidence of childhood obesity rise in Cameroon with a six-fold risk of developing hypertension in an obese child [17,28], the association between WC, WHtR and BMI with hypertension in children and adolescents in our setting has not been adequately explored. This study therefore set out to determine the proportion of secondary school adolescents with elevated BP and high blood pressure in relation to some measures of adiposity (BMI, WC, WHtR) and to examine the association between blood pressure and the adiposity indices among these children in the Bamenda municipality of the North West Region of Cameroon.

## Methods

**Study participants:** this study made use of an institution-based data collected between March 2022 to June 2022, involving children and adolescents aged 10 -19 years attending some secondary schools in the North West Region (NWR) of Cameroon selected through a 2-stage sampling technique. The first stage of the recruitment process was a random selection of 4 secondary schools (2 public and 2 private) from the list of 66 secondary schools which was obtained from the Regional Delegation of Secondary Education of the NWR of Cameroon and asked to take part in our study. During the second stage, a quota sampling technique was used to select a total of 602 children and adolescents from the different levels of study in secondary education. However, 68 of the children and adolescents were excluded from the study during data analysis given that they were underweight and had incomplete data giving a final dataset of 534 children and adolescents (168 boys and 366 girls) with mean age 15.1 ± 2.3 years. Using a prevalence of 21.6% obtained from a study by Srirama and Subramanian in India, amongst school children and a level of significance (ɑ) of 5%, a minimum sample size of 328 children and adolescents was obtained using the Cochran's formula [29].

### Data collection

**Anthropometric measurements:** all anthro pometric measurements (weight, height and waist circumference) were taken by well-trained nurses during school hours (9.00am and 1.00pm) on the school campuses ensuring all standard protocols were respected. Height was measured without shoes using a portable stadiometer (Seca 213, Germany), to the nearest 0.1cm. Body weight was measured to the nearest 0.1kg using a digital scale (Omron BF511, Japan). The BMI (body mass index) for each child was calculated by dividing weight (kg) by height (cm) squared [30]. Waist circumference was measured to the nearest 0.5cm using an inelastic and flexible ergonomic circumference tape (Seca 201, USA), according to the protocol by McCarthy *et al*. [31]. Waist-to-height ratio was also used to assess central obesity. The WHtR was calculated by dividing the WC (cm) by height (cm) and all the children were classified as ´low risk´ and ´high risk´ of developing cardiovascular diseases when the WHtR was < 0.5 and ≥ 0.5 respectively [26].

**Blood pressure:** systolic and diastolic blood pressure of the children were measured using an automated blood pressure device (SANITAS SBM21, Hamburg, Germany). Blood pressure was measured with the children sitting in a relaxed position with the arm resting and the palm facing upwards on the same day, three times within a 3-minute interval. The average of the three (3) measurements was recorded. Elevated BP and hypertension were defined as three elevated systolic or diastolic BP readings of ≥ 90^th^ percentile to <95^th^ percentile and ≥ 95^th^ percentile (for the child's age, sex and height) respectively [32].

**Statistical analysis:** IBM-SPSS for Windows version 23 statistical package was used for data analysis. Normality of all continuous variables was checked using the Kolmogorov-Smirnov (K-S) test. Weight, height, BMI and WC were adjusted for age and gender (z scores) using the WHO LMS Growth software [33]. This package has different growth reference data including WHO (2006), WHO (2007), UK-CDC (2000) and the British (1990) growth reference data for children and adolescents. The study participants were classified as being overweight (>1 z-score) and obese (> 2 z-score) with respect to BMI. Also, a WC z-score of 1.33 was used to classify participants as overweight/obese [34]. In addition, a WHtR of ≥ 0.5 was used to define participants at high cardiometabolic risk (“high risk”) [26]. The prevalence of elevated BP and hypertension for the different adiposity indices (BMI, WC and WHtR) were then calculated. The association between categorical variables was assessed using Chi-square test and proportions presented with their respective 95% confidence intervals. In addition, the means of continuous variables was assessed using an independent student t-test and ANOVA as appropriate. Pearson correlation was used to assess the association between the measures of adiposity with blood pressure (SBP and DBP). Finally, linear regression models (unadjusted and adjusted for age, gender and school type) were used to assess the relationship between the adiposity indices (BMI, WC, WHtR) with blood pressure (SBP and DBP). A p-value of < 0.05 was set as cut off for statistical significance.

**Ethical considerations:** the approval for this study was obtained from The University of Bamenda`s Institutional Review Board (IRB) (Ref: 2022/0421H/UBa/IRB). Administrative authorization was obtained from the Regional Delegation of Public Health of the North West Region and Regional Delegation for Basic Education of the North West Region (Ref. No. G649/1188/MINSEC/RDSE/NW/SDGA of 13 Jan 2022). In addition, all the principals/parents/guardians gave written informed consent was given by all the children/adolescents before any data collection procedure was carried out.

## Results

**Descriptive characteristics of the study population:** Table 1 is a description of the study population by gender. On average males were significantly (p <0.001) older and taller than females. Also, we observed that 68.7% of females were BMI-obese compared to 13.3% of males and this difference was significant (p= 0.001). In addition, females had a higher mean diastolic BP (68.3mmHg) compared to males (64.5 mmHg) and this difference was significant (p= 0.001). However, there was no significant difference (p >0.05) in the mean body weight, WHtR, WC and systolic BP between males and females. In addition, a higher proportion of females (23.2%) were BMI-overweight/obese compared to males (11.3%) and the difference was significant (X^2^=10.403, p= 0.001). With respect to WC, it was observed that a significantly (p <0.001) higher proportion of girls (36.1%) were centrally overweight/obese (WC) compared to boys (4.2%). While, the proportion of females with WHtR ≥ 0.5 was higher (3.8%) compared to males (1.8%). However, it was not significant (X^2^=1.721, p =0.291).

**Table 1:** characteristics of the study participants by gender

Variable	Whole sample n=534	Males (n=168)	Females (n=366)	P-value
	Mean (SD)	Mean (SD)	Mean (SD)	
Age (years)	15.1 (2.3)	15.7 (2.3)	14.9(2.2)	<0.001
Height (cm)	159.3 (11.0)	163.7 (13.3)	157.3(9.2)	<0.001
Height z-score+	-0.37 (1.4)	-0.61 (1.5)	-0.26(1.4)	0.011
Weight (kg)	58.4 (10.2)	59.6(10.7)	57.9(10.2)	0.087
Weight z-score+	0.57 (0.95)	0.21(0.92)	0.73(0.93)	<0.001
BMI (kg/m^2^)	22.9 (3.5)	22.2 (3.6)	23.3(3.4)	<0.002
BMI z-score+	0.87 (0.83)	0.62 (0.84)	0.98(0.81)	<0.001
WHtR	0.37 (0.06)	0.37(0.05)	0.37(0.06)	0.258
Waist circumference (cm)	69.9 (7.2)	69.2(6.6)	70.3(7.4)	0.092
WC z-score+	0.56(1.2)	-0.10(0.86)	0.87(1.1)	<0.001
Systolic BP (mmHg)	116.4(15.7)	115.6(15.0)	116.7(16.0)	0.416
Diastolic BP (mmHg)	67.1(11.9)	64.5(12.7)	68.3(11.3)	0.001

+Based on WHO 2007 reference data; BMI: body mass index; WHtR:waist-to-height ratio; BP: blood pressure;WC:waist circumference

**Figure 1:**
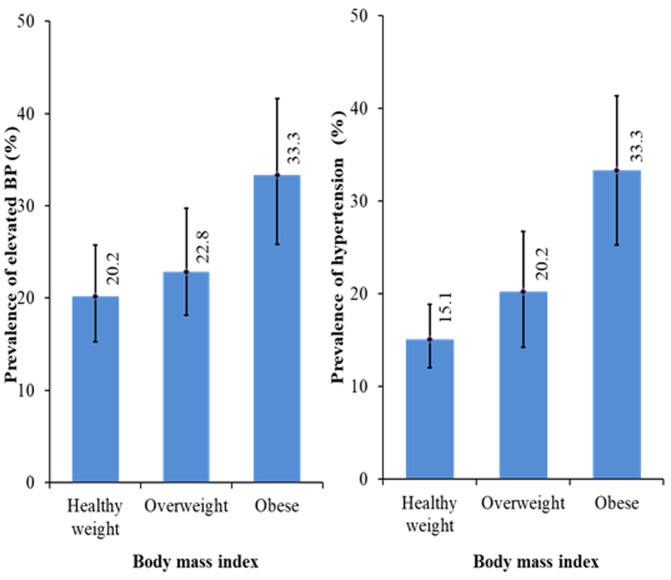
prevalence of elevated blood pressure and hypertension in the study population with respect to Body mass index (BMI)

**Figure 2:**
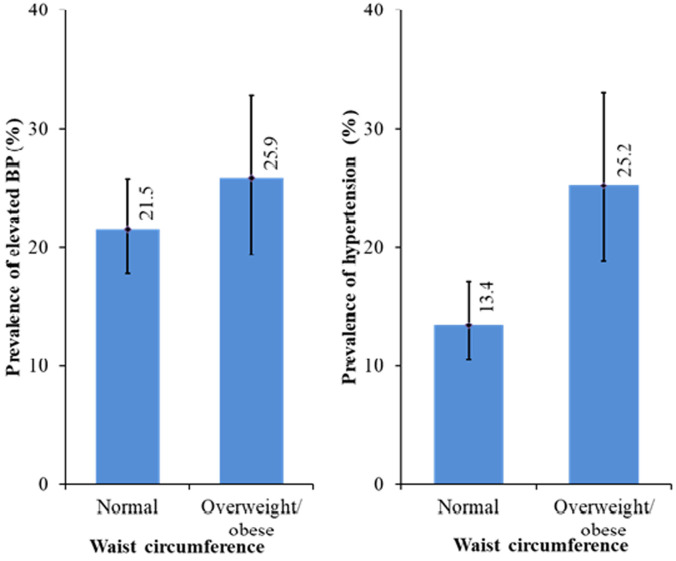
prevalence of elevated blood pressure and hypertension in the study population with respect to waist circumference (WC)

**Figure 3:**
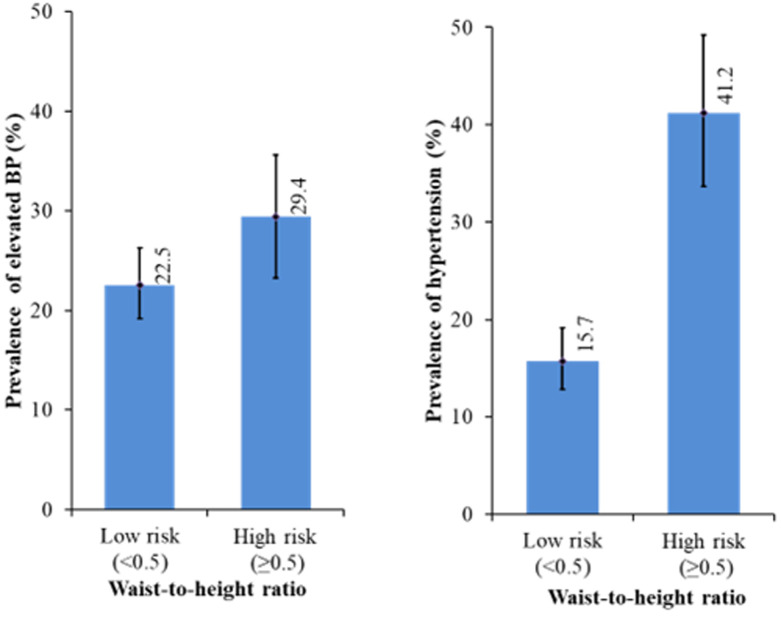
prevalence of elevated blood pressure and hypertension in the study population with respect to waist-to-height ratio (WHtR)

**Prevalence of elevated blood pressure and hypertension:** the overall prevalence of elevated BP and hypertension in this study was 39.2% (with 8.6% and 7.9% of the hypertensive children in stage I and stage II respectively) (Table 2, Table 3). With respect to gender, there was a non-significant difference (X^2^=0.474, p =0.765) in the proportion of females with elevated BP and high BP compared to males (39.9% vs 37.5%). However, more hypertensive females (8.5%) were in Stage II compared to males (6.5%). Figure 1, Figure 2 and Figure 3 shows the prevalence of elevated BP and hypertension by BMI, WC and WHtR amongst the study participants. A higher proportion of the BMI overweight/obese children had elevated BP (56.1%) and high blood pressure (53.5%) compared to their healthy weight counterparts (20.2% and 15.1%) respectively. However, the difference was not significant (X^2^= 6.818, p =0.146). Also, it was observed that a higher proportion of the centrally overweight/obese children (WC) had elevated BP (25.9%) and hypertension (25.2%) compared to children with normal WC (21.5% and 14.3%) respectively and the difference was significant (X^2^= 15.452, p <0.001). In addition, there was a significant difference (X^2^= 9.585, p = 0.002) in the proportion of ‘high-risk´ children (WHtR ≥ 0.5) with elevated BP (29.4%) and hypertension (41.2%) compared to the “low-risk” (WHtR < 0.5) children (22.4% and 15.4%) respectively.

**Table 2:** prevalence of elevated blood pressure and HTN amongst the study participants

Blood pressure status	N	Frequency	
		%	(95% CI)
Normal blood pressure	325	60.9	(56.6 - 64.9)
Elevated blood pressure	121	22.7	(19.3 - 26.4)
Hypertension			
Stage I	46	8.6	(6.5 -11.3)
Stage II	42	7.9	(5.8 - 10.5)

HTN: hypertension; CI:confidence interval

**Table 3:** prevalence of elevated blood pressure and HTN amongst the study participants by gender

Variable	All the children (N=534)	Males (N= 168)		Females (N=366)		p-value
	n (%)	n (%)	(95% CI)	n (%)	(95% CI)	
Blood pressure status						0.765
Normotensive	325 (60.9)	105(62.5)	(56.9 - 67.5)	220(60.1)	(55.0 - 65.0)	
Elevated BP	121(22.7)	35(20.8)	(15.4 - 27.6)	86(23.5)	(19.4 - 28.1)	
Hypertensive	88(16.5)	28(16.7)	(13.4 - 26.4)	60(16.4)	(12.9 -20.5)	

BP: blood pressure; HTN: hypertension

**Mean blood pressure profile of study participants according to weight status:** Table 4 shows the mean blood pressure profile of study participants according to weight status. Considering the whole sample, there was on average a 17.1mmHg and 9.4mmHg significant (p < 0.05) differences in the mean SBP and DBP observed amongst the BMI-obese children and the healthy weight children respectively. Similarly, there was a 13.9mmHg and 4.6mmHg significant (p <0.05) differences in the mean SBP and DBP between children who were centrally overweight/obese (WC) and those with normal WC respectively. Additionally, we observed a significant (p <0.05) difference in the mean SBP and DBP amongst children whose WHtR was ≥ 0.5 and < 0.5 respectively. With respect to gender, we observed a 19.1mmHg and 9.8mmHg significant (p < 0.05) mean differences in SBP and DBP between the BMI-obese females and the BMI healthy weight females respectively. Similarly, a 15.8mmHg and 9.5mmHg significant (p < 0.05) differences were also seen in the mean SBP and DBP between the centrally overweight/obese (WC) females and the normal weight females respectively. Furthermore, there was a significantly (p =0.007) higher mean SBP (136.2mmHg) amongst the WHtR “high risk” females compared to their “low risk” counterparts (115.9mmHg). On the contrary, there was a 9.5mmHg non-significant ((p = 0.051) difference in the mean DBP between the WHtR “high risk” females compared to the “low risk” group. Table 5 shows the Pearson correlation coefficients between the different measures of adiposity with blood pressure (SBP and DBP). In this present study, we found a significant positive weak correlation (p<0.001) between BMI (r= 0.169), WC (r= 0.329) and WHtR (r= 0.237) with systolic BP. Similarly, a significant positive weak association (p < 0.05) was observed between the BMI (r = 0.089), WC (r= 0.118) and WHtR (r= 0.095) with diastolic BP.

**Table 4:** mean blood pressure profile of study participants according to weight status

Variables		Blood pressure status					p-value
		SBP (mmHg)		p-value	DBP (mmHg)		
	N	Mean	(95% CI)		Mean	(95% CI)	
**Whole sample**							
BMI category (kg/m^2^)				<0.001*			0.004*
Healthy weight	430	115.4	(113.9 -116.9)		66.4	(65.3 - 67.6)	
Overweight	89	118.2	(115.1-121.4)		68.7	(66.5 - 70.9)	
Obese	15	132.5	(119.6 -145.3)		75.8	(66.7 - 84.9)	
Waist circumference(cm)				<0.001**			0.022**
Normal weight	495	115.4	(114.1-116.7)		66.7	(65.7 - 67.7)	
Central overweight/obesity	39	129.3	(123.5- 135.1)		71.3	(67.1 - 75.5)	
WHtR				0.004**			0.038**
Low risk (< 0.5)	517	115.8	(114.5- 117.1)		66.8	(65.8 - 67.8)	
High risk (≥ 0.05)	17	133.9	(122.4 -145.4)		75.4	(67.4 - 83.4)	
**Males**							
BMI category (kg/m^2^)				0.711*			0.986*
Healthy weight	149	115.2	(112.7 -117.8)		64.4	(62.4 -66.6)	
Overweight	17	118.2	(113.5 -122.9)		64.5	(59.1 - 70.0)	
Obese	2	118.5	(102.4 -162.9)		66.0	(62.6 - 66.4)	
Waist circumference (cm)							
Normal	158	115.1	(112.7 -117.5)	0.081*	64.5	(62.6 - 66.6)	0.702**
Central overweight/obesity	10	123.6	114.2 -133.0)		63.0	(56.1 - 69.9)	
WHtR				0.367 **			0.836**
Low risk (< 0.5)	165	115.4	(113.1-117.7)		64.5	(66.8 - 69.0)	
High risk (≥ 0.05)	3	123.3	(100.7-145.7)		66.0	(67.9 - 86.9)	
**Females**							
BMI category (kg/m^2^)				<0.001*			0.004*
Healthy weight	281	115.5	(113.8-117.3)		67.5	(66.2 -68.8)	
Overweight	72	118.2	(114.5 –121.9)		69.7	(67.3 - 72.1)	
Obese	13	134.6	(119.9 –149.3)		77.3	(58.2 - 94.8)	
Waist circumference (cm)				<0.001**			0.004**
Normal	337	115.5	(113.9 -117.1)		67.8	(66.6 - 68.9)	
Central overweight/obesity	29	131.3	(124.0 -138.6)		74.1	(69.2 --79.0)	
WHtR				0.007 **			0.051**
Low risk (< 0.5)	352	115.9	(114.3- 117.5)		67.9	(66.8 - 69.0)	
High risk (≥ 0.05)	14	136.2	(122.4 -149.9)		77.4	(67.9 - 86.9)	

* Calculated using one way analysis of variance (ANOVA); ** Calculated using student t-test; WHtR: Waist to Height; SBP: systolic blood pressure; DBP: diastolic blood pressure;WC: waist circumference

**Table 5:** Pearson correlation between blood pressure and measures of adiposity

Measures of adiposity	Pearson’s coefficient (r)	p-value
**Systolic blood pressure**		
BMI (kg/m^2^)	0.169	<0.001
WC (cm)	0.329	<0.001
WHtR	0.237	<0.001
**Diastolic blood pressure**		
BMI (kg/m^2^)	0.089	0.040
WC (cm)	0.118	0.007
WHtR	0.095	0.029

BMI: body mass index;WC: waist circumference WHtR: waist-to-height ratio

**Association between the measures of adiposity and blood pressure (SBP and DBP):** Table 6 shows the linear regression (unadjusted and adjusted for age, gender and school type) for the association between BMI, WC, WHtR and blood pressure amongst the study participants. There was a significant positive association (p <0.001) between waist circumference (β= 0.75; 95% CI= 0.57, 0.92), BMI (β= 0.88; 95% CI= 0.49, 1.25) and WHtR (β= 67.08; 95% CI= 45.64, 88.51) with systolic blood pressure for the unadjusted analysis. After adjusting for age, gender and school type, only waist circumference (β= 0.66; 95% CI = (0.43, 0.89) was significantly associated positively (p<0.001) with systolic blood pressure. Similarly, there was a significant positive association (p <0.003) between waist circumference (β= 0.24; 95% CI= 0.10, 0.38), BMI (β= 0.44; 95% CI= 0.15, 0.73) and WHtR (β= 25.19; 95% CI= 8.54, 41.86) with diastolic blood pressure for the unadjusted analysis. However, after adjusting for age, gender and school type, there was no significant association (p >0.05) between the measures of adiposity (BMI, WC and WHtR) with diastolic blood pressure.

**Table 6:** linear regression analysis (unadjusted and adjusted) for the association between the measures of adiposity and blood pressure among participants

Exposure	Unadjusted			Adjusted for age, gender and school type		
	Estimate (β)	(95% CI)	p-value	Estimate (β)	(95% CI)	p-value
**Systolic blood pressure (mmHg)**						
Waist circumference (cm)	0.75	(0.57, 0.92)	<0.001	0.66	(0.43, 0.89)	**<0.001**
BMI (kg/m^2^)	0.88	(0.49, 1.25)	<0.001	- 0.34	(-0.97, 0.29)	0.293
Waist-to-height (WHtR) ratio	67.08	(45.64, 88.51)	<0.001	34.25	(-6.88, 54.75)	0.102
**Diastolic blood pressure (mmHg)**						
Waist circumference (cm)	0.24	(0.10, 0.38)	0.001	0.16	(-0.02, 0.34)	0.081
BMI (kg/m^2^)	0.44	(0.15, 0.73)	0.003	0.11	(-0.39, 0.61)	0.662
Waist-to-height (WHtR) ratio	25.19	(8.54, 41.86)	0.003	3.04	(-29.48, 35.56)	0.854

BMI: body mass index

## Discussion

Measures of adiposity such as BMI, WC and WHtR have been shown to be associated with high BP in children and adolescents. However, very little attention has been focused on the influence of these different anthropometric indices of obesity on blood pressure in the paediatric population in spite of the fact that these measures increase the risk of high BP even in BMI-healthy weight children [35,36]. Early routine diagnosis, management and control of high BP through the periodic evaluation of anthropometric indices in children and adolescents especially in normal weight children is essential for the prevention of childhood obesity-related cardiovascular outcomes. This study set out to determine the proportion of secondary school adolescents with elevated BP and high blood pressure in relation to some measures of obesity (BMI, WC, WHtR) and to examine the association between blood pressure and the obesity indices (BMI, WC, WHtR) among these children in the Bamenda municipality of the North West Region of Cameroon. This study found that the prevalence of elevated BP and hypertension amongst the study participants was high for the BMI-obese, WC overweight/obese children and the “high risk” (WHtR ≥ 0.5) children and adolescents respectively. In addition, this study found that BMI, WC and WHtR were positively associated with blood pressure with waist circumference being an independent predictor for blood pressure in the children and adolescents in our setting. This study found that the overall prevalence of elevated BP and hypertension amongst the children was 39.2% (with 8.6% and 7.9% of the hypertensive children in Stage I and Stage II respectively). In Cameroon, there is limited population data on paediatric hypertension and adiposity. The findings of our study are higher than those reported by Noubiap *et al*. [12] in a systematic review in sub-Saharan Africa, where they reported an overall prevalence of 5.5% for elevated BP amongst children and adolescents. The findings are also higher than those reported in the Centre region of Cameroon by Chelo *et al*. [14] who found a prevalence of 1.6%.

However, the study in the Centre region was carried out on primary school children aged 5-17 years. Again, in Malaysia [37] amongst secondary school children aged 13 -15 years, the prevalence of elevated BP and hypertension were 13.2% and 17.0% respectively, lower than the values reported in our study. Similarly in Nigeria a survey by Okpokowuruk and colleagues [38] in a semi-urban environment reported a hypertension prevalence of 2.6%, lower than that reported in our study. However, the findings in our study are similar to those reported in Dar-es-Salaam in Tanzania by Muhihi *et al*. [39] who reported a hypertension prevalence of 15.2% in primary school children 5- 17 years and 19.2% in Pakistan [40] where a hypertension prevalence of 19.2% was reported. However, the Pakistani study was carried out on children 4-12 years old who had not attained puberty. Methodological differences, including the variations in statistical methods may explain the discrepancies in the prevalence of hypertension in our study with that observed in the other studies. Moreover, there has been evidence of an overweight/obesity and nutrition transition in school-aged children in sub-Saharan Africa and the changes in dietary and lifestyle patterns differs across the countries in the African region as such may explain the differences in the prevalence of hypertension amongst school going children and adolescents between our study and that of other studies observed across the continent. In addition, considering the whole sample, we found that the prevalence of hypertension was 53.6%, 25.2% and 41.2% amongst the BMI overweight/obese, centrally overweight/obese (WC) and the children with an increased cardiometabolic risk (WHtR ≥ 0.5) respectively. These findings are higher than those reported in Brazil [41] and China [42] where the prevalence of elevated BP using central overweight/obesity (WC) was 18.0% and 13.2% but similar to those obtained in Tanzania [39] and Pakistan [40] where a prevalence of 15.2% and 19.2% were reported respectively. These findings highlight the necessity for cost-effective strategies for creating awareness tailored to our context, early diagnosis, control and proper management of children with elevated BP in order to prevent in order to reduce the complications resulting from hypertension early in childhood and in later life.

The high prevalence of hypertension from the different adiposity indices in our study participants might be attributed to the obesity epidemic combined with the fact that adolescence is a period of independence with less parental control compounded with lifestyle challenges such as low levels of physical activity, unhealthy diet and socio-cultural factors, all of which increase the risk of hypertension. In addition, the higher proportion in girls compared to boys might be due to hormonal changes and psychosocial factors resulting from the rapid biological changes associated with puberty onset in this age group. Our findings highlight the need for periodic screening of secondary school children and adolescents including those with BMI-healthy weight using all the adiposity indices for the early identification of incident HTN in this vulnerable population thereby preventing adverse cardiovascular outcomes in early life. Evidence suggest that obese children and adolescents have an increased carotid artery intima-media thickness than their non-obese participants [21]. Our study found that 19.5% of the investigated children were BMI-overweight/obese, 26% were centrally overweight/obese (WC) and 3.2% were at “high risk” (WHtR ≥ 0.5). These findings are in higher than those reported amongst secondary school children in China [42] and Malaysia [37] where the prevalence of overweight/ obesity was 18.3% and 24.3% respectively. The differences in the prevalence of central overweight/obesity might be attributed to the small sample size in our study compared to the Chinese and Malaysian studies which involved 29,516 and 2,461 secondary school children and adolescents respectively. Evidence has proven that in the children and adolescents, the risk of developing hypertension in an overweight/obese child is six-times higher compared to a child with normal BMI [12]. Also, it has been shown that children and adolescents at “high risk” (WHtR ≥ 0.5) have an increased risk of developing high BP levels [43]. Our study found that the BMI-obese, centrally overweight/obese (WC) and “high risk” (WHtR ≥ 0.5) children had a significantly higher mean systolic BP and diastolic BP compared to their normal weight counterparts. In addition, the BMI-obese, centrally overweight/obese (WC) and “high risk” (WHtR ≥ 0.5) girls had a significantly higher mean systolic BP and diastolic BP compared to their normal weight or “low risk” counterparts, a finding which was not observed in males.

These findings are in contrast to those obtained in children and adolescents in Lithuania [13] and China [42] who found that boys had a significantly higher mean SBP and DBP compared to girls. The differences in blood pressure by gender can be attributed to sex hormones which have been found to be strongly involved in the developmental differences in blood pressure between men and women with BP progressing more rapidly in women than in men beginning early in life [44]. Again, this study revealed that BMI, WC and WHtR which are all measures of obesity were significantly and positively correlated with systolic BP and diastolic BP. These findings are similar to those reported by Kuciene *et al*. [13] amongst Lithuanian children and adolescents where they also reported a positive correlation between BMI, WC and WHtR with systolic and diastolic BP. Similarly, studies amongst Malaysian [45] and Brazilian [46] secondary school children and adolescents also found that BMI, WC and WHtR were significantly associated with hypertension in the paediatric population and could be used as indicators for children and adolescents at risk of hypertension. Visceral fat measured using waist circumference has been found to be strongly correlated with blood pressure [45]. This present study found WC as an independent predictor for children and adolescents at risk of hypertension. This finding is in line with findings in other countries where WC has also been found to be significantly associated with high BP in the paediatric population [46,47]. This finding suggests that amongst children and adolescents in our setting, WC can be used as a screening tool for children and adolescent at risk of hypertension. This finding highlights the necessity for more efforts on health promotion in schools by encouraging regular physical activity, healthy nutrition and weight control in the paediatric population as a primary prevention method for elevated BP in this vulnerable group.

The limitations to this study worth mentioning include: firstly, the tanner staging to assess the level of puberty in the children and adolescents was not done and this could have affected the relationship between WC, BMI and WHtR with blood pressure especially in females. Also, the influence of genetics on BMI cannot be completely ruled out given that family history of hypertension and obesity were not assessed in this present study. In addition, given that malaria can result in an enlarged spleen, the spleen size of the children was not measured in this present study as such it might have influenced the WC values. Finally, this study was carried out only in one municipality in one region of the country as such the findings might not truly be representative of the blood pressure profile of all secondary school adolescents in the country. Despite the limitations of this study, the study is innovative in that it is the first study in Cameroon to the best of our knowledge describing the association between three measures of obesity (BMI, WC and WHtR) with blood pressure amongst secondary school adolescents in the North West Region of Cameroon.

## Conclusion

This study conducted among secondary school adolescents in Cameroon found that the overall prevalence of elevated BP and hypertension amongst the study participants was high (39.2%). In addition, the study has demonstrated that WC is positively associated with high BP in children and adolescents. Therefore, WC can be used in predicting children and adolescents at high risk of hypertension in early life in our setting, given that centrally overweight/obese children at risk of hypertension might be missed if BMI is the only measure for adiposity. Further research needs to be conducted in other regions of the country to assess the relationship between WC and a positive family history with other risk factors for cardiovascular diseases in children and adolescents in order to reduce the risk of cardiovascular diseases in later life.

### What is known about this topic

- *Overweight and obesity using body mass index as a proxy for overweight and obesity is linked with pediatric hypertension;*
- *Pubertal hypertension is a strong predictor for adult hypertension or hypertension in later life 3;*
- *Height has been positively associated with childhood hypertension*.

### What this study adds

- *The prevalence of elevated blood pressure and hypertension amongst the children was high;*
- *Body mass index, waist circumference (WC) and waist-to-height ratio (WHtR were positively correlated with blood pressure in this study and it’s the first in Cameroon to use three indices of adiposity amongst secondary school BMI- healthy weight adolescents to assess its relationship with hypertension;*
- *Waist circumference was found to be an independent predictor for hypertension in our setting*.
